# Clinical Outcomes of “Paralyzed” Nerve Transfer for Treating Spinal Cord Injury: A Proof of Concept in a Human Model

**DOI:** 10.7759/cureus.52447

**Published:** 2024-01-17

**Authors:** Kyle J Chepla, Blake Perkins, Anne M Bryden, Michael W Keith

**Affiliations:** 1 Surgery, MetroHealth Medical Center, Cleveland, USA; 2 Physical Medicine and Rehabilitation, MetroHealth Medical Center, Cleveland, USA; 3 Orthopaedic Surgery, MetroHealth Medical Center, Cleveland, USA

**Keywords:** peripheral nerve transfers, hand reconstructive surgery, functional electrical stimulation, nerve surgery, spinal cord injury

## Abstract

Functional electrical stimulation (FES) is an option to restore function in individuals after high cervical spinal cord injury (SCI) who have limited available options for tendon or nerve transfer. To be considered for FES implantation, patients must possess upper motor neuron (UMN) type denervation in potential recipient muscles, which can be confirmed by response to surface electrical stimulation during clinical evaluation. Lower motor neuron (LMN) denervated muscles will not respond to electrical stimulation and, therefore, are unavailable for use in an FES system. Previous animal studies have demonstrated that a “paralyzed” nerve transfer of a UMN-denervated motor branch to an LMN-denervated motor branch can restore electrical excitability in the recipient. In this study, we report the indications, surgical technique, and successful outcome (restoration of M3 elbow flexion) after the first “paralyzed” nerve transfer in a human patient.

## Introduction

Functional electrical stimulation (FES) for patients with tetraplegia after spinal cord injury (SCI) has demonstrated positive outcomes and benefits regarding independence and the ability to perform activities of daily living [1]. Continuing advances make it an attractive option for patients with high cervical injury for which traditional nerve and tendon transfers are not possible [2,3]. Muscles innervated by cervical root levels below and outside the zone of SCI have an associated upper motor neuron (UMN) injury and will remain responsive to stimulation indefinitely. This is in contrast to muscles innervated by cervical roots that originate within the zone of injury and have lower motor neuron (LMN) involvement, which renders them non-responsive to stimulation and therefore unavailable for incorporation into an FES system [4]. A previous animal study demonstrated that the transfer of “paralyzed” (non-volitional) UMN peripheral nerves from below the zone of injury to (non-volitional) LMN nerves within the zone of injury can restore electrical excitability [5]. In clinical practice, this could be used to increase the number of muscles available for current FES systems and improve functional outcomes. We hypothesize that this will be successful in humans and present outcomes after paralyzed nerve transfers for the restoration of stimulated elbow flexion.

## Case presentation

A 28-year-old male presented six months after a gunshot wound to the neck with a complete (ASIA A) C4 level injury. His trapezius strength was 5/5, graded by the British Medical Research Council (BMRC), but no upper extremity muscles demonstrated volitional activity (0/5 BMRC). He had limited passive external rotation of the shoulder to neutral, with no contractures present.

In our tetraplegia management clinic, we routinely use surface-stimulated manual muscle testing (SMMT) graded using the BMRC scale to differentiate between UMN (SMMT BMRC 4-5) and LMN (SMMT BMRC 0-1) involvement in potential recipients for nerve transfer after SCI [4]. The evaluation of this patient showed no response to surface stimulation, consistent with LMN injury in the supraspinatus, infraspinatus, deltoid, and biceps (SMMT 0/5). SMMT of the triceps (5/5), flexor carpi radialis (FCR) and flexor carpi ulnaris (FCU) (4/5), and extensor carpi radialis brevis (ECRB) (4/5) indicated UMN injury. Therefore, we identified these as potential donors for time-dependent paralyzed transfer.

The patient underwent paralyzed nerve transfers in the right upper extremity nine months after the injury. This included transferring the medial head of the triceps to the axillary nerve, fascicles of the FCR and FCU to the brachialis and biceps, respectively, in the upper arm, and a spinal accessory nerve (SAN) to suprascapular nerve (SSN) transfer, all to restore electrical excitability in the recipient muscles and active rotator cuff and shoulder motion.

In the immediate post-operative period, the patient was put into a light compressive wrap and sling to minimize edema and protect the transfer sites. He was cleared to resume normal hygiene and return to his previous activity level on post-operative day five, with instructions to avoid direct pressure over the nerve transfer sites for two to three weeks. Despite limited published evidence, sensory and motor rehabilitation and re-education are considered integral to achieving optimal outcomes after nerve transfer surgery [6]. Early-phase rehabilitation for this patient included protected range-of-motion exercises to prevent joint stiffness and improve a pre-existing internal rotation contracture. Additionally, efforts were made to maintain passive range of motion and joint excursion within the planes and directions of motion to be restored by the nerve transfer.

One month post-operatively, the patient began a home exercise program including habitual active firing of the upper trapezius to restore voluntary control of the supraspinatus and infraspinatus. He was provided with a Chattanooga Continuum portable neuromuscular electrical stimulation (NMES) unit (Enovis, Lewisville, Texas) and began home stimulation over the donor and recipient muscles using parameters reported by Javeed et al., aiming to preserve muscle mass and accelerate axonal outgrowth [7]. This was started at 30 minutes daily and progressed up to one hour if skin irritation, pain, and autonomic dysreflexia were not encountered.

Pairing of donor and recipient activation was performed until trace contraction of the recipient muscle was observed 12 months postoperatively. Late-phase rehabilitation, which focuses on unpairing donor activation and transitions to strengthening and engaging plasticity mechanisms in peripheral neural pathways, was then started [8,9]. At two years post-operatively, the patient achieved 90-degree anti-gravity flexion of the biceps with surface stimulation (Video 1). With gravity eliminated, >100 degrees of elbow flexion was possible with surface stimulation (SMMT 4/5) and, when used in conjunction with a Bioness Wireless Hand Rehabilitation System (Bioventus, Durham, NC), the patient was able to grasp and bring items towards his mouth with shoulder support (Video 2).

**Video 1:** Post-operative stimulated elbow flexion This video taken two years postoperatively demonstrates restoration of electrical excitability and anti-gravity elbow flexion in response to surface stimulation after "paralyzed" transfer.

**Video 2:** Surface stimulation in conjunction with Bioness FES to restore elbow flexion and grasp This video demonstrates how the restoration of stimulated elbow flexion can be combined with other FES modalities to potentially restore the ability to perform activities of daily living.

## Discussion

This case demonstrates that restoration of electrical excitability after “paralyzed” nerve transfer is possible in humans. Despite the restoration of electrical excitability in the biceps, no active shoulder motion was obtained from the spinal accessory nerve transfer, and surface stimulation of the deltoid was not possible postoperatively. The failure of the spinal accessory transfer might have been related to involvement of the suprascapular nerve distal to the transfer site due to the mechanism of injury. The failure of the deltoid to respond to stimulation may indicate an inadequate post-surgical rehabilitation protocol or a technical failure. Unlike traditional nerve transfers, where recipient muscles constantly receive consistent neural input post-operatively, paralyzed transfers rely on surface stimulation to generate new neural pathways, overcome atrophy, and decrease fatiguability. Increasing the frequency and length of stimulation over the deltoid may have resulted in improved clinical outcomes.

Despite this, the ability to restore electrical excitability in critical muscle groups, such as elbow flexion, creates exciting new opportunities for improving outcomes in individuals with high cervical SCI and for incorporation into new FES systems. Before demonstrating that paralyzed nerve transfers are possible, our group previously utilized stimulation of “paralyzed” (UMN injury) tendon transfers to restore functional deficits in muscles with an associated LMN injury. However, as with traditional tendon transfers, this requires the sacrifice of a potential donor that could be used for another function. Thus, the ability to restore, and increase, the number of stimulable muscles present in the upper extremity might improve outcomes with FES systems and warrants further study. Additionally, future studies focusing on indications, refining patient selection, and improving postoperative rehabilitation programs are needed and ongoing. The authors acknowledge that we are reporting outcomes on a single patient. However, we believe this technique should be reproducible based on previous animal studies and may offer exciting new reconstructive opportunities for patients with high cervical level injury and tetraplegia.

## Conclusions

This case report demonstrates that paralyzed nerve transfer can be successfully used to restore electrical excitability in human patients with high cervical SCI. This surgical technique can increase the number of stimulable muscles available for incorporation into FES systems, thereby enhancing system functionality and patient outcomes.
